# Printable Zinc-Ion Hybrid Micro-Capacitors for Flexible Self-Powered Integrated Units

**DOI:** 10.1007/s40820-020-00546-7

**Published:** 2020-11-05

**Authors:** Juan Zeng, Liubing Dong, Lulu Sun, Wen Wang, Yinhua Zhou, Lu Wei, Xin Guo

**Affiliations:** 1 State Key Laboratory of Material Processing and Die & Mould Technology, School of Materials Science and Engineering, Huazhong University of Science and Technology, Wuhan, 430074 People’s Republic of China; 2 College of Chemistry and Materials Science, Jinan University, Guangzhou, 511443 People’s Republic of China; 3 Wuhan National Laboratory for Optoelectronics, School of Optical and Electronic Information, Huazhong University of Science and Technology, Wuhan, 430074 People’s Republic of China

**Keywords:** Zinc-ion hybrid capacitor, Kelp-carbon, Zinc metal anode, Multivalent ion storage, Self-powered unit

## Abstract

Wearable self-powered systems integrated with energy conversion and storage devices such as solar-charging power units arouse widespread concerns in scientific and industrial realms. However, their applications are hampered by the restrictions of unbefitting size matching between integrated modules, limited tolerance to the variation of input current, reliability, and safety issues. Herein, flexible solar-charging self-powered units based on printed Zn-ion hybrid micro-capacitor as the energy storage module is developed. Unique 3D micro-/nano-architecture of the biomass kelp-carbon combined with multivalent ion (Zn^2+^) storage endows the aqueous Zn-ion hybrid capacitor with high specific capacity (196.7 mAh g^−1^ at 0.1 A g^−1^). By employing an in-plane asymmetric printing technique, the fabricated quasi-solid-state Zn-ion hybrid micro-capacitors exhibit high rate, long life and energy density up to 8.2 μWh cm^−2^. After integrating the micro-capacitor with organic solar cells, the derived self-powered system presents outstanding energy conversion/storage efficiency (*η*_overall_ = 17.8%), solar-charging cyclic stability (95% after 100 cycles), wide current tolerance, and good mechanical flexibility. Such portable, wearable, and green integrated units offer new insights into design of advanced self-powered systems toward the goal of developing highly safe, economic, stable, and long-life smart wearable electronics.

**Electronic supplementary material:**

The online version of this article (10.1007/s40820-020-00546-7) contains supplementary material, which is available to authorized users.

## Highlights

- This work is a new guide for the design of on-chip energy integrated systems toward the goal of developing highly safe, economic, and long-life smart wearable electronics.
- The biomass kelp-carbon based on unique 3D micro-/nanostructure combined with multivalent ion storage contributes to high capacity of the Zn-ion hybrid capacitor.
- The flexible solar-charging self-powered system with printed Zn-ion hybrid micro-capacitor as energy storage module exhibits fast photoelectric conversion/storage rate, good mechanical robustness, and cyclic stability.

**Electronic supplementary material:**

The online version of this article (10.1007/s40820-020-00546-7) contains supplementary material, which is available to authorized users.

## Graphical abstract

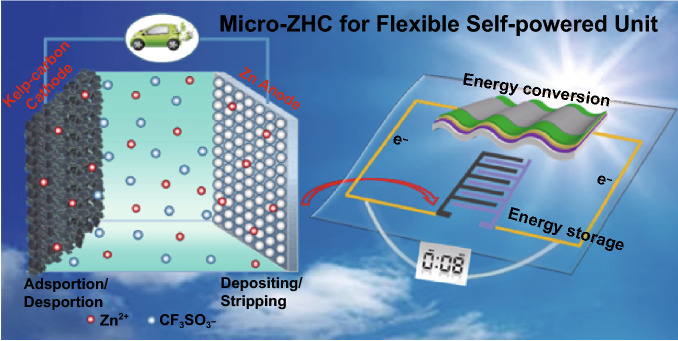

## Introduction

Wearable electronics need to be miniature, portable, highly integrated, and conformable to human skin or other tissues [1, 2]. Various human–machine interfaces, mobile power supplies, and display devices are expected to be integrated as multifunctional wearable systems, which would significantly improve the quality of our lives [3–5]. To build an independent functional wearable system, an energy conversion/harvesting–storing module serving as a power supply is prerequisite to other functional system components [6, 7].

In terms of energy conversion module, photovoltaic devices that convert solar energy to electric energy are omnipresent for harnessing clean and massive solar energy [8]. Yet solar energy is intermittent, unpredictable, and available only during the daytime. The unstable output energy is incapable of powering functional system persistently. Consequently, energy requires to be stored in a trustworthy module for subsequent use. Among numerous burgeoning energy storage devices, supercapacitors with high power density, fast charge-discharge rate, particularly the satisfying tolerance to variation of input current, have attracted great attention [9, 10]. Following characteristics make them very suitable to store the converted solar energy by photovoltaic devices to build self-powered systems: (i) two devices (*i.e.,* photovoltaic device and supercapacitor) complement each other. Photovoltaic devices continuously power the supercapacitors to resolve the shortcoming of low energy density for supercapacitors; the converted energy by photovoltaic devices is stored by the supercapacitor for use to resolve its shortcoming of intermittent source; (ii) it is a green energy system that does not need extra charging equipment, which has referential significance to other energy integration systems.

Supercapacitors suffer from relatively low energy density compared with various batteries, hindering their practical applications in advanced wearable electronics. Thus, to improve energy density is an eternal research objective for supercapacitors, and promising strategies care: (i) increasing the capacitance by introducing pseudocapacitive electrode materials; (ii) enhancing cell voltage by using novel electrolytes or constructing asymmetric devices [11–13]. Hybrid capacitors (a type of asymmetric supercapacitors) with one battery-type electrode as energy source and one capacitive electrode as power source are double-benefited [14, 15]. Various univalent metal ion hybrid capacitors, such as lithium-ion hybrid capacitors, sodium-ion hybrid capacitors, and potassium-ion hybrid capacitors, have been studied [16]. Nevertheless, alkali metals (Li, Na, and K) are extremely reactive, and introduced organic electrolytes are flammable, causing security risks. In recent years, multivalent ion storage mechanisms (*e.g.*, Zn^2+^, Mg^2+^, Ca^2+^, and Al^3+^) have been put forward, which may provide fast charge transfer dynamics, high capacity, and energy density [17]. Among them, Zn-ion hybrid capacitors (ZHCs) are considered as research hotspot by virtue of their unique merits including high safety, low-cost, high capacity, and long cycle life [18, 19]. Safety is a main concern for wearable electronic devices. Exploiting ZHCs based on quasi-solid-state aqueous electrolyte can surmount the hazards of flammability and electrolyte leakage, while improve energy density.

To match wearable electronics, power sources with traits of miniature, planar, reliable, and easy to integrate are demanded [20]. Micro-ZHCs based on planar interdigital structure can meet the above requirements. However, the studies on asymmetric micro-devices are still in their nascent stage owing to the difficulty in building micro-asymmetric configuration and processing with different electrode materials. To the best of our knowledge, solar-charging self-powered system based on micro-ZHCs as the energy storage module has not been reported yet.

Herein, we fabricate aqueous ZHCs and quasi-solid-state micro-ZHCs, targeting flexible solar-charging self-powered system. The aqueous ZHCs are constructed with distinctive biomass kelp-carbon as cathode, Zn foil as anode and zinc trifluoromethane sulfonate [Zn(CF_3_SO_3_)_2_] aqueous solution as electrolyte. The unique 3D micro-/nano- architecture of the kelp-carbon enables a high-rate and long-life ZHC; the asymmetric cell structure and multivalent ion (Zn^2+^) storage result in a high specific capacity and energy density of the device. Further, flexible quasi-solid-state micro-ZHCs based on the screen printed kelp-carbon cathode, Zn powder anode and Zn(CF_3_SO_3_)_2_/polyacrylamide (PAM) hydrogel electrolyte are fabricated on polyimide (PI) substrate. Screen printing technique is considered as a universal approach and applicable for scaled-up fabrication, which has the prospect of commercialization and gets rid of the cost dilemma faced by wearable self-powered devices in practical applications. The fabricated micro-ZHCs possess the virtues of integrated planar interdigital structure, excellent electrochemical performances, low cost, and high security. Moreover, direct printing such micro-ZHCs on chip with flexible organic solar cells (OSCs) enables the construction of solar-charging self-powered units with simplified configurations and mechanical robustness. The derived integrated system exhibits fast photoelectric conversion/storage characteristics, wide current tolerance, continuous self-powering and wearable features, demonstrating potential for applications in healthcare, human-machine interfaces, and intelligent robotics.

## Experimental Section

### Materials Preparation

#### Preparation of Kelp-Carbon

The preparation method of kelp-carbon refers to our previous work [21]. Kelp blades (for food) were purchased from local farmers' market. Before using, they were washed with deionized water and dried at 70 °C overnight. For carbonization, the kelp-blades were heated to 600 °C for 2 h under Ar atmosphere in a tube furnace. For activation, the carbonaceous precursor was ground into powders and then impregnated in KOH solution with a mass ratio of 1:4 (carbon: KOH). The mixed slurry was heat-treated at 120 °C for 24 h in a vacuum oven and then was heated to 800 °C for 3 h under Ar flow in a tube furnace. Afterward, the activated samples were thoroughly washed with 10 wt% HCl solution and deionized water, until neutral pH was achieved. After drying, kelp-carbon was obtained. Compared with our previous work [21], the main difference in the preparation process lies in the activation temperature for the kelp-carbon. Higher activation temperature benefits to widen the pore size of the activated carbon.

#### ***Preparation of Zn(CF***_***3***_***SO***_***3***_***)***_***2***_***-PAM Hydrogel Electrolyte***

Acrylamide monomer powders (4.2 g) were dissolved in deionized water (21 mL) with stirring for 0.5 h to obtain a uniform solution. Then, N,N'-methylenebisacrylamide (0.01 wt%), N,N,N',N'-tetramethylethylenediamine (0.09 wt%), and ammonium persulfate (0.1 wt%) were added into the solution and stirred uniformly. After degassing by ultrasonic treatment and vacuum, the obtained solution was poured into a glass mold and heated at 60 °C for 8 h. Afterward, the as-prepared polymer gel was soaked in 2 M Zn(CF_3_SO_3_)_2_ aqueous solution for more than 24 h to reach the ion balance point.

### Devices Preparation

#### Assembly of Aqueous ZHCs

Aqueous ZHCs were assembled using a typical coin cell structure (CR2016), with kelp-carbon as cathode material and Zn foil (thickness of 70 μm) as anode. The cathode was prepared by coating electrode slurry (the mass ratio of kelp-carbon/PVDF was 9:1) on stainless foil. The specific mass loading of the cathode material was 0.57–1.13 mg cm^−2^, the thickness of the cathode was ~ 0.02 mm, and the diameter of the electrode was 10 mm. 2 M Zn(CF_3_SO_3_)_2_ aqueous solution was used as the electrolyte (with a pH value of ~ 5.5), and commercial polypropylene film (MPF30, NKK) was utilized as the separator. For control experiment, ZHCs based on commercial activated carbon as cathode [TF-B520//2 M Zn(CF_3_SO_3_)_2_ //Zn] were also assembled.

#### Construction of Flexible Quasi-Solid-State Micro-ZHCs

Firstly, interdigital Au layer (200 nm in thickness) acting as current collector was patterned on flexible PI substrate with designed mask by using magnetron sputtering (Amod, Angstrom Engineering Inc.). Next, kelp-carbon paste (80% kelp-carbon and 20% organic vehicle composing of butyl carbitol, ethyl-cellulose, terpineol, din-butyl phthalates, and span85) was screen-printed (PHP-1515, Hotting Screen Printing Equipment Co. LTD) on the one side of the Au current collector fingers, and Zn paste (80% Zn powder and 20% organic vehicle) was screen-printed on the other side of the Au current collector fingers to fabricate the asymmetric microelectrodes. The printed micro-device was annealed at 200 °C for 2 h to remove the organic vehicle. Finally, a piece of Zn(CF_3_SO_3_)_2_-PAM hydrogel electrolyte was coated on the interdigital electrodes with copper tape employed as the wire lead, and encapsulated with polyethylene film (20 μm in thickness).

#### Preparation of Flexible OSCs

The detailed preparation process of flexible OSCs was provided in supporting information.

#### Construction of Flexible Solar-Charging Integrated Units

To deliver required working voltage, a micro-ZHC and four OSCs connected in series are integrated onto flexible polyethylene terephthalate (PET) substrate (110 μm in thickness) with copper foil tape as the interconnected wire. The active areas of the micro-ZHC and the four-junction OSCs are 0.0352 and 0.3 cm^2^, respectively. The entire device was sealed with polyethylene film.

### Materials and Characterizations

Materials and characterizations on the morphology, structure, and porosity of kelp-carbon, electrochemical measurements, and calculations are provided in supporting information.

## Results and Discussion

### Configuration of the Solar-Charging Self-Powered Unit

**Fig. 1:**
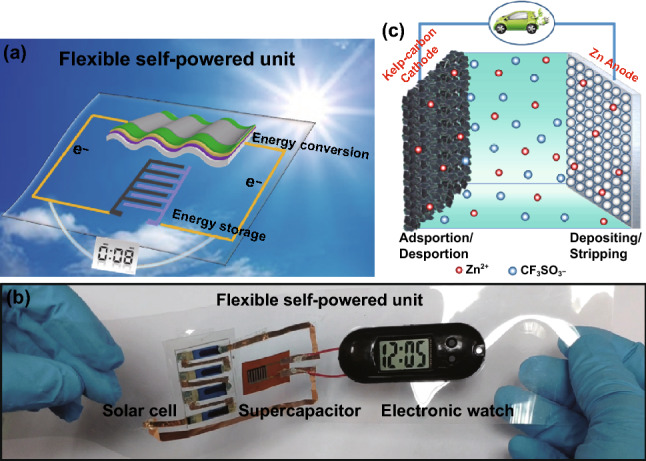
**a** Graphical illustration of the solar-charging self-powered unit. **b** Proof-of-concept demonstration of the flexible solar-charging self-powered unit. **c** Working mechanism of the developed ZHC

Figure 1a illustrates the configuration of the constructed flexible self-powered unit consisting of a piece of flexible PET substrate, energy conversion module (*i.e.,* flexible OSCs) and an energy storage module (*i.e.,* flexible micro-ZHC). Once the integrated unit is exposed to sunlight, the OSCs convert photo-irradiation into electricity and charge the micro-ZHC (solar-charging). The self-powered unit can power electronic devices during daylight, in doors and even at the time of discontinuous illumination or no light without external charging supply. As individual component is flexible and the entire integrated unit complies with an in-plane design, it endows the system high flexibility. Figure 1b shows a proof-of-concept demonstration, the integrated unit can function as a reliable power source to drive a portable electronic watch. The multivalent ion storage mechanism of the developed ZHC is presented in Fig. 1c. Highly reversible and fast ion (Zn^2+^ and CF_3_SO_3_^−^) adsorption/desorption storage mechanism occurs on the kelp-carbon cathode, Zn^2+^ depositing/stripping reaction occurs on the Zn anode, and Zn(CF_3_SO_3_)_2_ aqueous solution acts as the electrolyte.

### Characteristics of the Kelp-Carbon

**Fig. 2:**
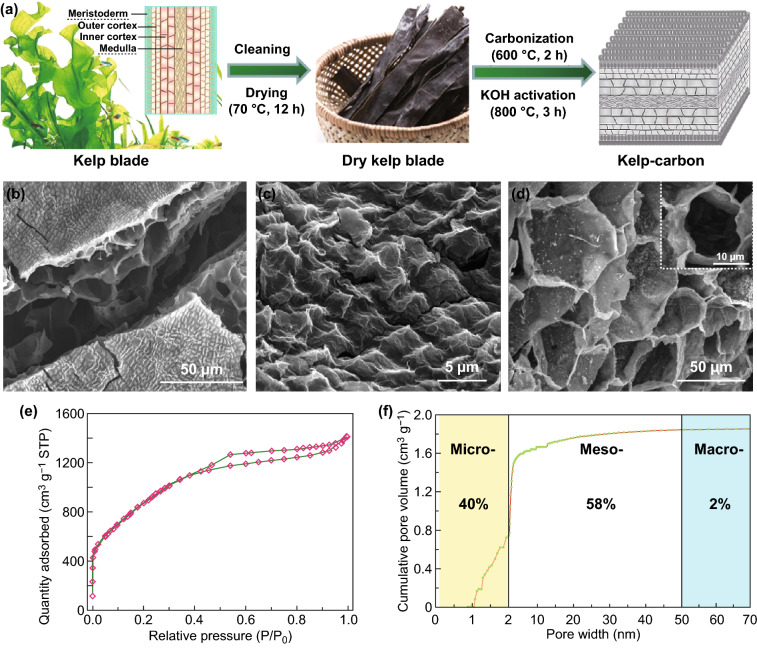
**a** Main preparation process of kelp-carbon, including graphical illustrations on the microstructure of kelp blade and 3D hierarchical architecture of kelp-carbon. SEM images of the kelp-carbon: **b** overall morphology, **c** surface morphology, and **d** interior morphology. **e** N_2_ adsorption/desorption isotherm and **f** 2D-NLDFT pore size distribution of the kelp-carbon

Kelp as a kind of brown algae is an extensive seafood worldwide. The biological structure of kelp blade is composed of meristoderm, outer cortex, inner cortex and medulla (Fig. 2a). To carry out effective photosynthesis in the sea, the surface layer (meristoderm) of kelp blade contains lots of chromatophores, which are made up of stacked nanolayered membranes and provide a high surface area. The cellulose and hemicellulose in the cell walls of the meristoderm, outer cortex, inner cortex, and medulla are pyrolyzed during carbonization, and form hierarchical, porous, and interconnected micro-/nano- architecture after activation (Fig. 2a, b)*.*

The kelp-carbon presents ridge-like surface morphology consisting of stacked carbon nanosheets (Fig. 2c), corresponding to the meristoderm cellularity in the surface layer of kelp blade. The interior of the kelp-carbon owns a 3D cellular-like architecture (Fig. 2b, d), which consists of quadrilateral/pentagon-like cells and cell walls (carbon nanosheets). The sizes of the polygonal cells rang from ten to dozens of microns. Transmission electron microscope (TEM), Raman spectroscopy, and X-ray diffraction (XRD) investigations indicate the disordered (amorphous) carbon microstructure of the kelp-carbon (Fig. S1). X-ray photoelectron spectroscopy (XPS) confirms the presence of C, O, N, S, and P elements in the kelp-carbon (Fig. S2a). The atomic fractions of C, O, N, S, and P are 88.81%, 9.89%, 0.8%, 0.46%, and 0.05%, respectively. The contents of N, S, and P elements are relatively low. The high-resolution C 1 s spectrum (Fig. S2b) exhibits strong *sp*^2^ carbon bonding (284.35 eV) and relatively weak C–O bonding (286.4 eV). The O 1 s spectrum (Fig. S2c) indicates the existence of a certain amount of oxygen-containing functional groups, including H_2_O–OH bonding (531.4 eV), adsorbed H_2_O molecules (532.5 eV) and C–O groups (533.8 eV). Brunauer–Emmett–Teller (BET)-specific surface area of the kelp-carbon reaches a high value of 3047 m^2^ g^−1^ according to N_2_ adsorption/desorption measurement (Fig. 2e). A hysteresis loop in the relative pressure range between 0.4 and 0.99 suggests the existence of mesopores and macropores. The pore size distribution curve (Fig. 2f) shows that the content of macropores is very low, micropores (0.74 cm^3^ g^−1^) and mesopores (1.1 cm^3^ g^−1^) occupying the most proportion. The total pore volume is as high as 1.87 cm^3^ g^−1^. The polygonal cell walls and quadrilateral/pentagon-like channels in the kelp-carbon could provide sufficient pathways for the fast electron and ion diffusion.

### Electrochemical Properties of the Aqueous ZHCs

**Fig. 3:**
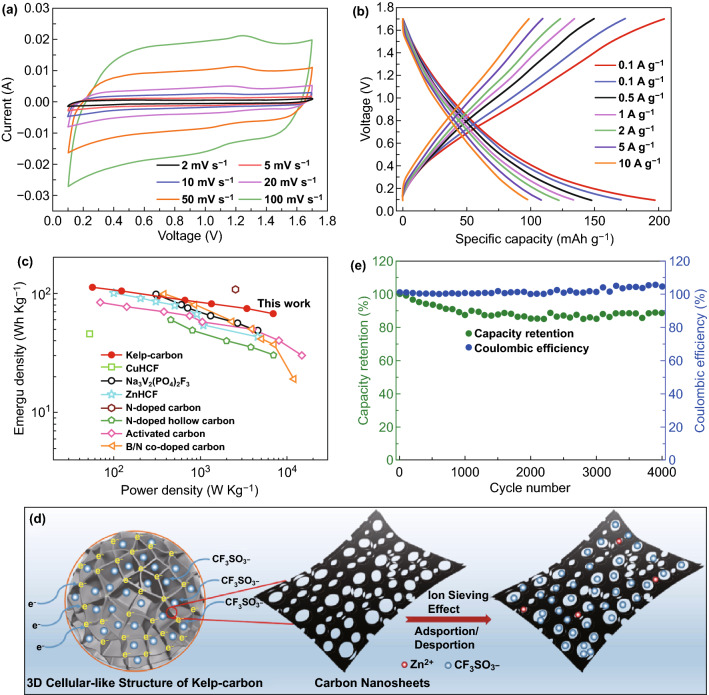
**a** CV curves at different scan rates, **b** GCD curves at different current densities, and **c** Ragone plots of the aqueous ZHC. Energy densities of other aqueous ZHCs and Zn-ion batteries reported in literatures are provided for comparison. **d** Schematic of the 3D porous structure of kelp-carbon and the ion sieving effect for carbon nanosheets in electrochemical process. **e** Cycling performance of the device at 2 A g^−1^

Figure 3a shows the cyclic voltammetry (CV) curves at various scan rates of the assembled aqueous ZHC (kelp-carbon//Zn(CF_3_SO_3_)_2_//Zn) in a cell voltage range of 0.1–1.7 V (in a wider voltage range, water decomposition may occur, Fig. S3) [22]. A pair of redox peaks located at 1.25 and 1.0 V are observed in the CV curves corresponding to the reactions of Zn/Zn^2+^ depositing/stripping on Zn anode [19]. The shapes of the CV curves and positions of the redox peaks do not distort obviously with increasing scan rates, manifesting good rate capability of the hybrid capacitor [23].

Galvanostatic charge–discharge (GCD) measurement was devoted to evaluate the specific capacity, energy density, and power density of the ZHC. The GCD curves (Fig. 3b) present basically symmetrical, and the maximum specific capacity (based on the mass of kelp-carbon) is calculated to be 196.7 mAh g^−1^ (445 F g^−1^ in specific capacitance) at 0.1 A g^−1^, and still holds 97.2 mAh g^−1^ (219 F g^−1^) at 10 A g^−1^. The maximum energy density of the ZHC is up to 111.5 Wh kg^−1^ at 1300 W kg^−1^. High power densities of 1.3–6.9 kW kg^−1^ within 35–219 s can be obtained for the cell, which are much higher than those of common zinc-ion batteries [24, 25]. It is worth mentioning that the specific capacity and energy density of our aqueous ZHCs are superior to those of the reported aqueous ZHCs based on carbon cathodes, such as commercial activated carbon [18], N-doped carbon [26], N-doped hollow carbon [27], and B/N-codoped carbon [28], and even precede those of some aqueous Zn-ion batteries [29–31]. Electrochemical impedance spectroscopy (Fig. S4a) reveals reasonable equivalent series resistance (*R*_*s *_*= *13 Ω) and charge transfer resistance (*R*_*ct *_*= *23.4 Ω) for the ZHC [26, 32], implying good conductivity of the electrode materials and fast transport of electrolyte ions in the electrode and at the interfaces between the electrode and electrolyte.

The specific capacity of ZHC is mainly determined by the cathode material (kelp-carbon). Activated carbons with partial micropores are recommended for enhancing ion-trapping [33], while a certain proportion of mesopores/macropores is prerequisite to reduce the ion diffusion resistance (especially for ions with relatively large ion radius) and could serve as reservoirs for electrolyte ions [21]. Pore structure is like a filter for electrolyte ions (*i.e.,* ion sieving effect) and the dimensions of pores and ions should match each other [33, 34]. As depicted in Fig. 2f, although the micropore sizes of the kelp-carbon (0.9–2 nm) are already larger than the hydrated ionic radii of CF_3_SO_3_^−^ ( > 0.58 nm) [35] and Zn^2+^(0.43 nm) [36]. In the actual ion adsorption/desorption process, a large number of bottleneck-type micropores generally exist in the activated carbons, which may block the rapid transport of electrolyte ions at high current densities and lead to poor rate capability [37]. Such phenomenon is announced from the control experiment by using commercial activated carbon (TF-B520) as cathode for the ZHCs. TF-B520 mainly contains micropores with an average pore size of 0.82 nm (Fig. S5). Due to the bottleneck effect of micropores and part of the micropores being underused, the ZHC based on TF-B520 cathode shows relatively low discharge-specific capacity (129 mAh g^−1^), worse rate performance, and higher *R*_ct_ (35 Ω) (Fig. S6).

The Warburg region of the Nyquist plots (Fig. S7a) was further analyzed by replotting *Z*′ (the real part of the collected impedance) as a function of *ω* ^−1/2^ (*ω* is the angular frequency) to reveal the ion diffusion resistivity in the kelp-carbon electrode. The slope of the linear fitting line is equal to diffusion resistivity, which reflects the ion diffusion impedance in the nanoporous carbons [38]. As shown in Fig. S7b, we can see that the slope of kelp-carbon is smaller than that of TF-B520, meaning reduced diffusion resistivity of ions in kelp-carbon electrode. Hence, we can conclude that the smaller *R*_ct_ and diffusion resistivity of kelp-carbon render its better rate capability performance (Figs. S4b and S6c). In our kelp-carbon, mesopores occupying the highest percentage (58%) contribute to the fast diffusion/transport of relatively large electrolyte ions (CF_3_SO_3_^−^ and Zn^2+^). Except for the hierarchical pore structure, owing to the interior 3D cellular-like geometry of the kelp-carbon, the interconnected cell walls and interpenetrating quadrilateral/pentagon-like channels are able to offer sufficient pathways for achieving rapid electron transfer and ion diffusion at the same time (Fig. 3d). The above characteristics are important for achieving high specific capacity and good power density. In addition, the working voltage window of our aqueous ZHC (1.6 V) is wider than that of common aqueous Zn-ion batteries (about 1 V) [39, 40]. High specific capacity combining with wide working voltage window contributes to the high energy densities of our ZHCs.

**Fig. 4:**
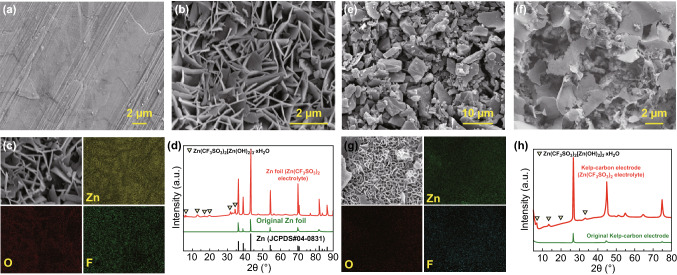
SEM images on the surface of Zn foil anode: **a** original and **b** after 4,000 cycles. **c** Corresponding EDS mapping on the surface of Zn foil anode after 4,000 cycles. **d** XRD patterns of the anode before and after 4,000 cycles. SEM images on the surfaces of kelp-carbon cathode: **e** original and **f** after 4,000 cycles. **g** Corresponding EDS mapping on the surface of kelp-carbon cathode after 4,000 cycles. **h** XRD patterns of the cathode before and after 4,000 cycles

Considering supercapacitors generally present better capacitance retention at high current densities due to the limitation of diffusion rate and limited reactions. Cycling stability of the aqueous ZHC was investigated at a moderate current density of 2 A g^−1^ (Fig. 3e), which may announce more realistic cyclic stability of the cell. The specific capacity of the ZHC retains 89% of its initial value, and the coulombic efficiency holds 105% after 4,000 charge/discharge cycling test. The coulombic efficiency above 100% is due to the hydrogen evolution reaction (electrolysis of water) on Zn anode during discharging. Hydrogen evolution consumes electrons and promotes the zinc anode oxidation and generates more electrons to reach the cutoff voltage of 0.1 V. Such reaction may contributes extra discharge capacity [41]. The reduction of capacity retention indicates some negative side-reactions occurred in the cycling process. In the mild acid electrolyte [2 M Zn(CF_3_SO_3_)_2_ aqueous solution with a pH value of ~ 5.5], side-reactions for the aqueous ZHCs are mainly related to the inevitable hydrogen evolution reaction on Zn anode and the generation of poorly conductive by-products on electrodes. After 4,000 cycles, plate-like Zn on Zn foil anode (Fig. 4a, b) and some thin nanosheets on the surface of kelp-carbon cathode (Fig. 4e, f) are observed. However, no obvious Zn-dendrites generates on Zn anode in the mild acid electrolyte, because Zn-dendrites problem generally happens in alkaline electrolyte [42]. Energy-dispersive spectrometer (EDS) mapping (Fig. 4c, d) and XRD analysis (Fig. 4g, h) reveal that by-product of Zn(CF_3_SO_3_)_2_[Zn(OH)_2_]_3_·xH_2_O generated on both electrodes [43, 44]. During discharging, Zn^2+^ cations strip from the Zn anode and are adsorbed on the porous kelp-carbon cathode; during charging, Zn^2+^ cations deposit on the Zn anode and CF_3_SO_3_^−^ anions are adsorbed on the kelp-carbon cathode. Zn^2+^ cations shuttle between the two electrodes. The precipitation/dissolution of Zn(CF_3_SO_3_)_2_[Zn(OH)_2_]_3_·xH_2_O participates forms according to reaction Eq. (1):

1 $$4{\text{Zn}}^{{2 + }} + {\text{ }}6{\text{OH}}^{ - } + {\text{ }}2\left( {{\text{CF}}_{3} {\text{SO}}_{3} } \right)^{ - } + {\text{ xH}}_{2} {\text{O}} \Leftrightarrow {\text{Zn}}\left( {{\text{CF}}_{3} {\text{SO}}_{3} } \right)_{2} \left[ {{\text{Zn}}\left( {{\text{OH}}} \right)_{2} } \right]_{3} \cdot {\text{xH}}_{2} {\text{O}} \downarrow$$

Existence of Zn(CF_3_SO_3_)_2_[Zn(OH)_2_]_3_·xH_2_O on both electrodes indicates enhanced pH value of the Zn(CF_3_SO_3_)_2_ electrolyte and generation of OH^−^ induced by hydrogen evolution reaction in our aqueous ZHCs, yet it is a common phenomenon in aqueous Zn-ion hybrid capacitors and Zn-ion batteries [18, 43]. The acceptable cycling stability of the aqueous ZHC at moderate current density benefits from the unique 3D hierarchical porous structure of the kelp-carbon and ample unimpeded pathways (interconnected cell walls and interpenetrating quadrilateral/pentagon-like channels) available for rapid electron and ion transport (Fig. 3d). Control experiment (Fig. S8) confirms that the microstructure of kelp-carbon is beneficial to alleviate the influence of by-product precipitations on cycling stability of the aqueous ZHC. When using TF-B520 as the cathode, the capacity retention of the aqueous ZHC reduces to only 49% after 4,000 cycles. Different from the 3D cellular-like geometry and hierarchical pore structure of kelp-carbon, commercial activated carbon TF-B520 has a solid and micropore structure. The micropores on the surface of TF-B520 can be easily blocked by by-product precipitations, and lack of open interpenetrating channels in TF-B520 results in poor cycling stability of the cell.

### Construction of Flexible Micro-ZHCs via Screen Printing

**Fig. 5:**
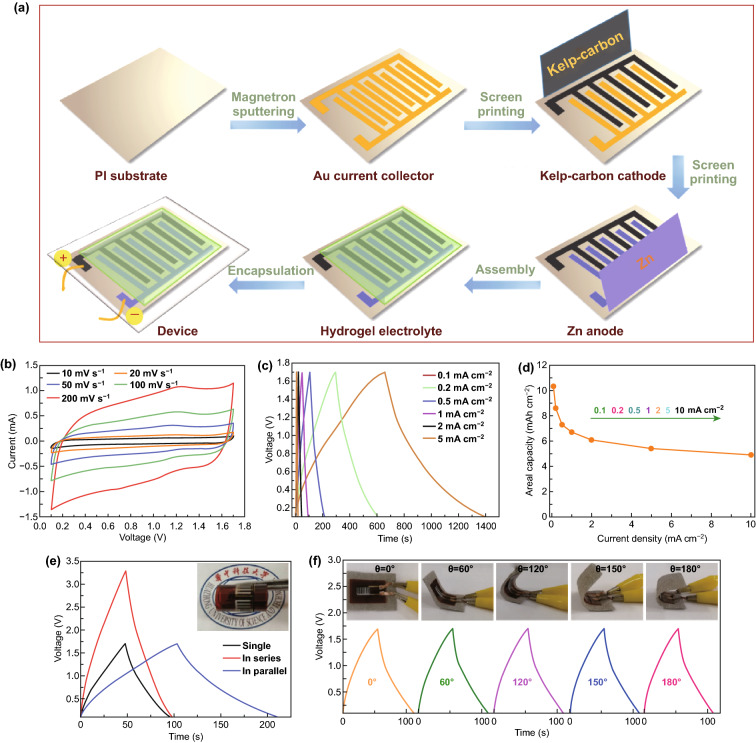
**a** Fabrication procedure for the flexible quasi-solid-state micro-ZHCs. **b** CV curves of the micro-ZHCs at different scan rates. **c** GCD curves at different current densities. **d** Areal capacity and rate capability. **e** GCD curves at 1 mA cm^−2^ of a single cell, and two cells connected in series or parallel. The inset shows two cells connected in series and good flexibility of the device. **f** GCD curves at 1 mA cm^−2^ of the micro-ZHCs under different bending angles from 0° to 180°

In quest to miniaturized, lightweight, and wearable energy storage devices, we developed flexible quasi-solid-state micro-ZHCs via a facile and economic screen-printing technique. Figure 5a describes the simple preparation process. The Au current collectors were deposited by magnetron sputtering, followed by screen printing kelp-carbon cathode and Zn powder anode, and coating the printed interdigital electrodes with Zn(CF_3_SO_3_)_2_-PAM hydrogel electrolyte finally. The crosslinked PAM polymer chains can form an ion conductive network with superb water absorption ability and flexibility, as exhibited in Fig. S9. The ionic conductivity of the Zn(CF_3_SO_3_)_2_-PAM hydrogel electrolyte is up to 12.2 mS cm^−1^, as determined by the electrochemical impedance test (Fig. S10). The surface morphology of the obtained interdigital electrodes after screen printing (printing one layer) is shown in Fig. S11a. The width of one electrode finger and interspace between the cathode and anode fingers are 400 and 250 μm, respectively (Fig. S11b, c). The thicknesses of the kelp-carbon cathode and the Zn powder anode determined by SEM are about 10 and 10.35 μm, respectively (Fig. S12).

To evaluate the electrochemical performances of the printed quasi-solid-state micro-ZHCs, CV and GCD measurements with a voltage range from 0.1 to 1.7 V were carried out. The CV curves (Fig. 5b) with a pair of redox peaks are in line with the results of Fig. 3a, attributed to the hybrid energy storage mechanism. Even at high scan rates, the quasi-rectangle shapes are well maintained, indicating excellent rate capability of the micro-ZHCs. Compared with the aforementioned stacked ZHCs, in-plane micro-ZHCs possess better rate capability due to the architecture of interdigital microelectrodes, which could reduce the ion transport path and make full use of the active materials. The GCD curves demonstrate symmetrical triangular shapes with no apparent internal resistance drop. The highest areal capacity of the device (based on the total area of positive and negative electrodes) reaches 10.28 μAh cm^−2^ at a current density of 0.1 mA cm^−2^ (Fig. 5c, d). The micro-ZHC has an areal energy density of 8.2 μWh cm^−2^ at a power density of 40 μW cm^−2^ and still maintain 3.9 μWh cm^−2^ at 4 mW cm^−2^. It is worth mentioning that we just print one layer of electrode materials on the Au current collectors. However, the thickness and mass loading of the active electrode material can be further increased by multiple printing [45, 46]. This is an advantage by using screen printing technology. Even so, the areal energy density of our micro-ZHC is comparable to those of the recently reported in-plane micro-supercapacitors based on carbon materials [47–51]. More information on the specific capacity/capacitance and energy/power density of the micro-ZHC based on different metrics (areal or volumetric performance) is summarized in Table S1 for cross-lab comparison.

Moreover, the voltage window or output current of the device can be enlarged by printing the electrode arrays in series or parallel to meet the energy/power requirement. Figures 5e and S13a show that the operating voltage and output current are readily doubled when two micro-ZHCs are connected in series and in parallel, respectively. In series connection, the voltage window reaches up to 3.2 V which is twice the value of a single ZHC, and there is a degradation in the output current due to the increased resistance. In parallel connection, the ZHCs provide nearly twice the output current of a single ZHC, while holding the same voltage window as that of the single cell. Owing to the flexibility of the microelectrodes and hydrogel electrolyte, the fabricated micro-ZHCs could subject to mechanical bending with maintained capacitive functionality. To evaluate the mechanical robustness of the printed micro-ZHC, the device was subjected to mechanical bending from 0° to 180° (Figs. 5f and S13b) and repeated bending at 120° for 100 cycles (Fig. S14), the CV and GCD curves resemble the ones under flat state with capacity retention close to 100%. The negligible effect of bending on the electrochemical properties of the micro-ZHC indicates good adhesion between Au current collector and electrodes. Such printed micro-ZHCs with impressive mechanical and electrochemical stabilities promise multi-field integration applications.

### Integration of Flexible Solar-Charging Self-Powered Units

**Fig. 6:**
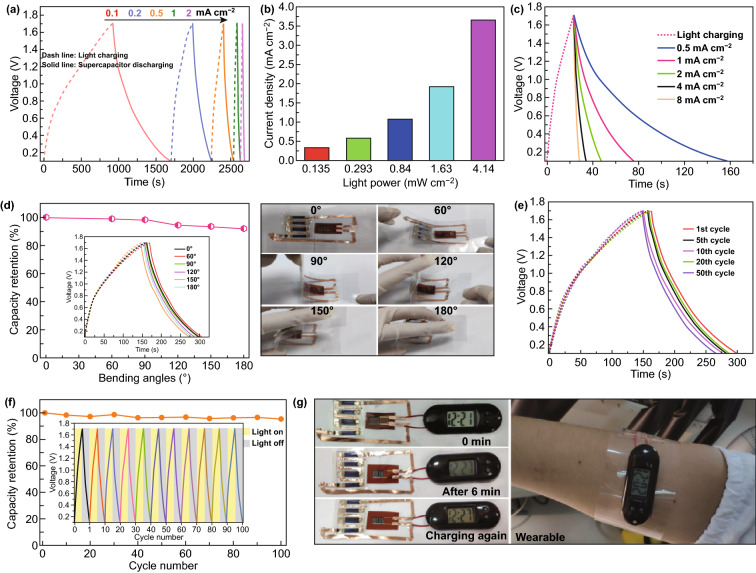
**a** Solar-charging/discharging curves of the integrated self-powered unit at different light intensities/discharge current densities. **b** Output current densities of the OSCs at different light intensities. **c** Solar-charging/discharging curves at the same light intensity and different discharge current densities. **d** Capacity retentions of the unit at different bending angles from 0° to 180° and corresponding GCD curves. **e** Cycling stability of the unit at a bending angle of 120° for 50 cycles. **f** Cycling performance of the unit at 1 mA cm^−2^. **g** Proof-of-concept demonstration of the flexible solar-charging self-powered unit powering an electronic watch

To build a solar-charging self-powered unit, micro-ZHC serving as the energy storage module and four-junction OSCs working as the energy conversion component are integrated on PET substrate. The performance and the photovoltaic parameters of the single flexible OSC under different light sources (AM 1.5G and LED) are provided in Fig. S15 and Table S2. The energy conversion efficiency of the OSC reaches 21.2% at the indoor light intensity (0.135 mW cm^−2^). Considering that the standard sunlight intensity is not always available (such as cloudy and rainy days), and we usually work and live in the indoor environment, it is very necessary to evaluate the performance of the self-powered integrated unit at weak light intensities. The solar-charging/discharging performance of the integrated system was evaluated under varied indoor light intensities (0.135–4.14 mW cm^−2^) with homologous discharge current density (Fig. 6a). The system worked well in a wide range of light intensities with no obvious IR drop observed in the solar-charging/discharging curves (thus low energy loss), and the charging time can be adjusted according to the light intensity. The output current density of the OSCs corresponding to light intensity is shown in Fig. 6b. The highest energy conversion/storage efficiency (*η*_overall_) of the integrated unit reached 17.8% at a light intensity of 0.135 mW cm^−2^. As shown in Fig. 6c, the integrated unit can be charged to 1.6 V in 23 s, demonstrating fast photoelectric conversion rate. Moreover, the integrated unit was discharged at different current densities from 0.5 to 8 mA cm^−2^, and the diploid discharge time implies a favorable rate performance.

We further investigated the mechanical stability of the integrated system. Figure 6d shows the solar-charging (0.84 mW cm^−2^)/discharging (0.5 mA cm^−2^) profiles at various bending angles (60°, 90°, 120°, 150°, and 180°). The roughly superposed charge/discharge curves demonstrate excellent flexibility and electrochemical stability of our self-powered unit. The mechanical durability was further explored by repeatedly bending the device at 120° for 50 cycles (Fig. 6e). The capacity retained 86% of its original value after 50 bending cycles, indicating decent mechanical stability of the self-powered unit. The cycling stability of the system was firstly investigated at a solar-charging intensity of 1.63 mW cm^−2^ and discharge current density of 1 mA cm^−2^ (Fig. 6f). Impressively, the system exhibits superb cycling stability with a capacity retention of 95% after 100 cycles. Then, we conducted the cycling test on the system at a higher solar-charging intensity of 4.14 mW cm^−2^ (which is the highest light intensity that our indoor light source, *i.e.,* LED lamp can provide) and discharge current density of 2 mA cm^−2^ (Fig. S16). The system presents a capacity retention of 91% after 100 cycles, slightly lower than the value at a solar-charging intensity of 1.63 mW cm^−1^ (95%) due to the higher charge–discharge current densities for the micro-ZHC. Considering the good flexibility and electrochemical stability of the self-powered unit, it is befitting to be applied in wearable scenarios. We utilized the flexible self-powered unit as a “wearable wristband” to power an electronic watch (1.5 V). The electronic watch could work for a long time when the wristband was exposed to indoor natural sunlight after fast charging at a light intensity of 12 mW cm^−2^ (Fig. 6g). Moreover, energy stored in the micro-ZHC could power the electronic watch for more than 6 min in dark, after that the system could return to normal work just by solar-charging again, demonstrating a green energy system.

## Conclusions

A low cost, safe, durable, and flexible solar-charging integrated unit is developed. The system consists of OSCs and a micro-ZHC acting as energy conversion and storage module, respectively. The in-plane asymmetric printing technology employed by micro-ZHCs is an economic, facile, and versatile fabrication method. The unique 3D hierarchical architecture of kelp-carbon and multivalent ion storage mechanism endow the micro-ZHCs with high areal capacity of 10.28 μAh cm^−2^ and high energy density of 8.2 μWh cm^−2^. The integrated unit exhibits fast photoelectric conversion characteristic (charged to 1.6 V in 23 s) with tolerance for a wide variation of light intensity (0.135–4.14 mW cm^−2^). It shows excellent mechanical robustness and cycling stability (with a capacity retention of 95% after 100 cycles). Moreover, the integrated unit can power an electronic watch easily under indoor natural light, demonstrating its wearability and practicality. Such portable, wearable, and green self-powered unit is believed to be a new guide for design of energy integrated systems toward the goal of developing highly safe, light weight, economic, and long-life smart wearable electronics.

## Electronic supplementary material

Below is the link to the electronic supplementary material.

Supplementary file1 (PDF 1400 kb)
