# Supplementary material for: Printable Zinc-Ion Hybrid Micro-Capacitors for Flexible Self-Powered Integrated Units
Source: Nanomicro Lett. 2020 Nov 5;13:19. doi: 10.1007/s40820-020-00546-7 (PMC8187672; doi:10.1007/s40820-020-00546-7)
Supplement: Supplementary file 1 — Supplementary file1 (PDF 1400 kb) [file 40820_2020_546_MOESM1_ESM.pdf]

Supporting Information for

Printable Zinc-Ion Hybrid Micro-Capacitors for Flexible Self-Powered Integrated Units

Juan Zeng[1], Liubing Dong[2], Lulu Sun[3], Wen Wang[3], Yinhua Zhou[3], Lu Wei[1,*], Xin Guo[1,*]

[1]State Key Laboratory of Material Processing and Die & Mould Technology, School of Materials Science and Engineering, Huazhong University of Science and Technology, Wuhan 430074, People's Republic of China

[2]College of Chemistry and Materials Science, Jinan University, Guangzhou 511443, People's Republic of China

[3]Wuhan National Laboratory for Optoelectronics, School of Optical and Electronic Information, Huazhong University of Science and Technology, Wuhan 430074, People's Republic of China

*Corresponding authors. E-mail: lwei@hust.edu.cn (Lu Wei); xguo@hust.edu.cn (Xin Guo)

S1 Experimental Section

S1.1 Materials

The polymer donor (PBDB-T-2F) and small molecule acceptor (IT-4F) were purchased from Organtecsolar Materials Inc. Zinc acetate dehydrate, ethanolamine and methoxyethanol were purchased from Sigma-Aldrich. The sol-gel ZnO solution was prepared by dissolving zinc acetate dihydrate (0.1 g) in methoxyethanol (1 mL) and ethanolamine (0.028 g).

S1.2 OSC fabrication

The structure of the flexible OSC is PET/ITO/ZnO/PBDB-T-2F:IT-4F/MoO_3/Ag. Isopropanol and acetone were used to clean the substrates. The electron transport layer ZnO was spun on the ITO/PET at 3500 rpm for 60 s and annealed at 130 °C for 15 min. Then, the PBDB-T-2F:IT-4F films were prepared on the top of ZnO by spin coating from a chlorobenzene solution (containing 0.5 vol% of 1,8-diiodooctane) with PBDB-T-2F:IT-4F (10 mg/mL: 10 mg/mL) at 1500 rpm for 60 s, and then annealed at 100 °C for 10 min in a glove box filled with nitrogen. Finally, MoO_3 (7 nm) and Ag (100 nm) were thermally evaporated on the active layer.

S1.3 Characterizations

SEM (Nova NanoSEM 450, FEI Corporation, Netherlands) equipped with EDS and TEM (Tecnai G2 F30, FEI Corporation, Netherlands) were used to observe the micro-morphologies of samples and analyze the element compositions. The microstructure of kelp-carbon was characterized by Raman spectroscopy using a LabRAM HR800 spectrometer (HORIBA Jobin Yvon Corporation, France) with a Nd:YAG laser at a wavelength of 532 nm. XRD (X'Pert

PRO, PANalytical B.V., Netherlands) was used to analyze the crystal structure of materials and electrodes. The element composition of the kelp-carbon was identified by XPS collected on an AXIS Ultra DLD-600W spectrometer (Kratos Corporation, Japan) with a monochromatic Al Kα X-ray source. N_2 adsorption/desorption measurements were carried out at 77 K using an ASAP 2020 surface area and porosity analyzer (Micromeritics Instrument Corporation, USA). Before testing, the samples were degassed at 350 °C overnight. CV tests were performed in the voltage range between 0.1 and 1.7 V at scan rates from 5 to 200 mV s^{-1} using a Gamry Interface 1000 electrochemical workstation. EIS measurements were executed in the frequency range from 10^{-3} to 10^5 Hz at open circuit potential with an AC amplitude of 5 mV. GCD tests were carried out on Arbin BT 2000 Battery Testing System in the voltage window of 0.1 to 1.7 V at current densities from 0.1 to 10 A g^{-1}. The cycle stability tests were performed at 2 A g^{-1}.

S1.4 Calculations

The specific capacity (C_m, mAh g^{-1}) for the ZHCs can be calculated from discharge curve by Eq. (S1):

$$C_m = \frac{It}{m} \tag{S1}$$

where I (mA), t (h) represent the current and time of discharge process, and m (g) stands for the mass of cathode material.

Energy density (E, Wh kg^{-1}) and power density (P, W kg^{-1}) for the ZHCs were calculated by Eqs. (S2) and (S3), respectively:

$$E = C_m * U \tag{S2}$$

$$P = \frac{E}{t} \tag{S3}$$

where U (V) and t (h) stand for the discharge voltage after ohmic drop and discharge time.

The areal capacity (C_A, mAh cm^{-2}) of the micro-ZHCs can be calculated from GCD curves *via* Eq. (S4):

$$C_A = \frac{It}{A} \tag{S4}$$

where I (mA) and t (h) represent the current and time of discharge process, and A (cm^2) stand for the total area of cathode and anode.

The energy density E_A (mWh cm^{-2}) and power density P_A (mW cm^{-2}) were obtained from the Eqs. (S5) and (S6), respectively:

$$E_A = C_A * U \tag{S5}$$

$$P_A = \frac{E_A}{t} \tag{S6}$$

where U (V) and t (h) stand for the discharge voltage after ohmic drop and discharge time.

The overall efficiency of energy conversion and storage (η_{overall}) can be calculated according to Eq. (S7):

$$\eta_{overall} = \frac{E}{P \times S \times t} \qquad \text{(S7)}$$

where *E*, *P*, *S,* and *t* are the discharge energy after solar-charging (Wh), the light intensity (W m^{-2}), the effective area of solar cell (m^2), and the duration of solar-charging (h), respectively.

S2 Supplementary Figures and Tables

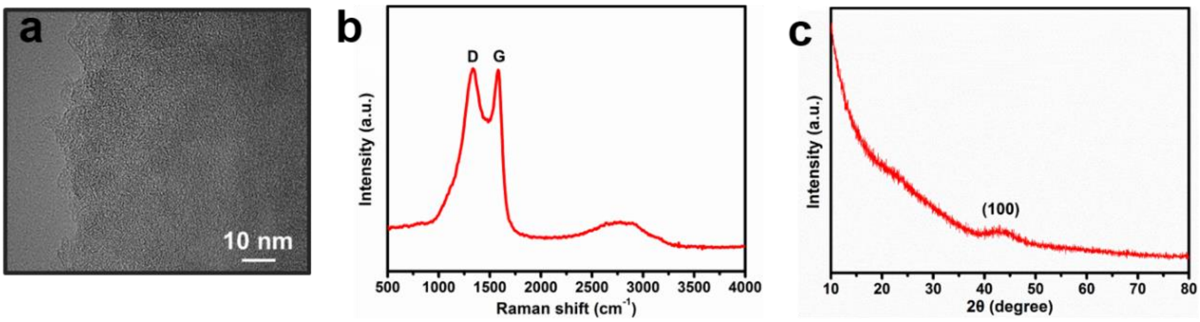

Fig. S1 (**a**) TEM image, (**b**) Raman spectrum and (**c**) XRD pattern of the kelp-carbon

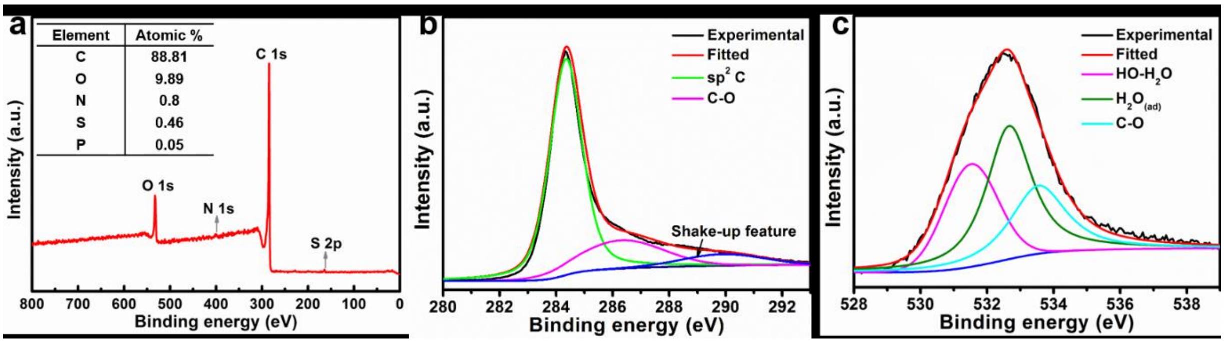

Fig. S2 (**a**) XPS spectrum, (**b**) high-resolution C 1s and (**c**) high-resolution O 1s spectra of kelp-carbon

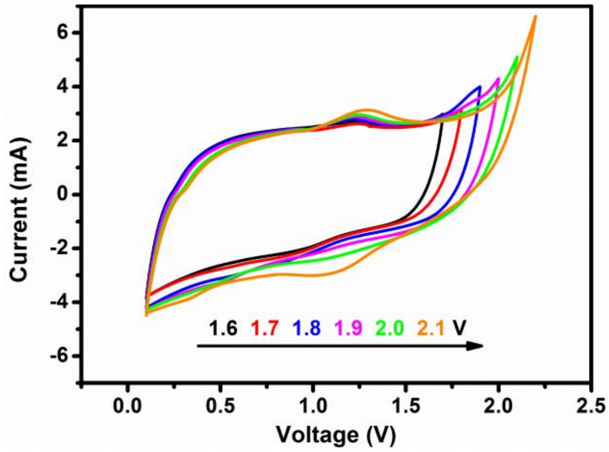

Fig. S3 CV curves of the aqueous ZHC in operating voltage window from 1.6 to 2.1 V at a scan rate of 10 mV s^{-1}

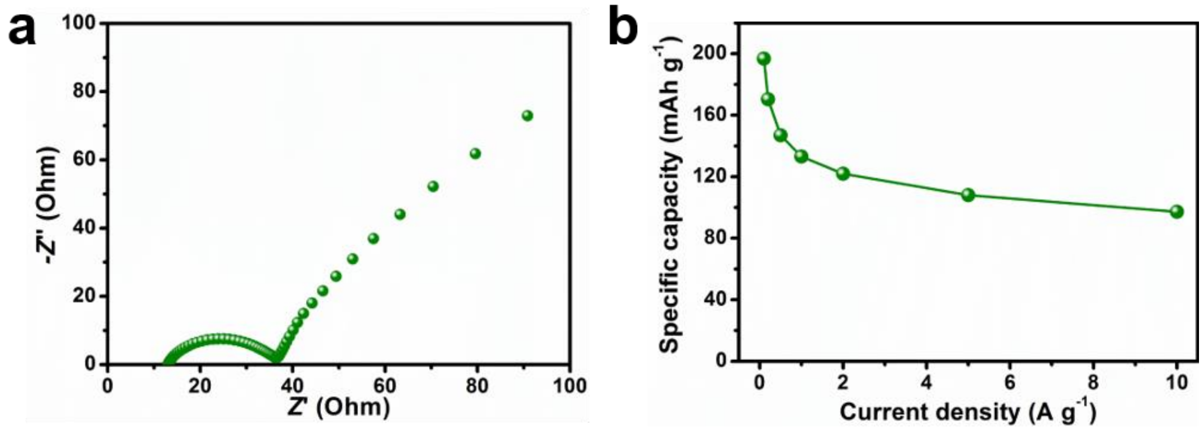

Fig. S4 (**a**) Nyquist plot and (**b**) rate capability of the aqueous ZHC

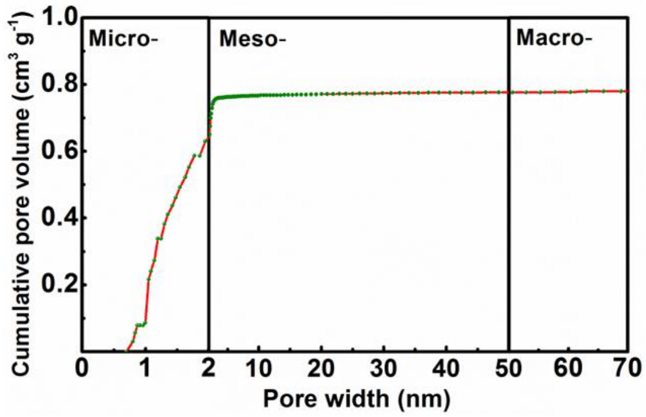

Fig. S5 2D-NLDFT pore size distribution curve for the commercial activated carbon (TF-B520)

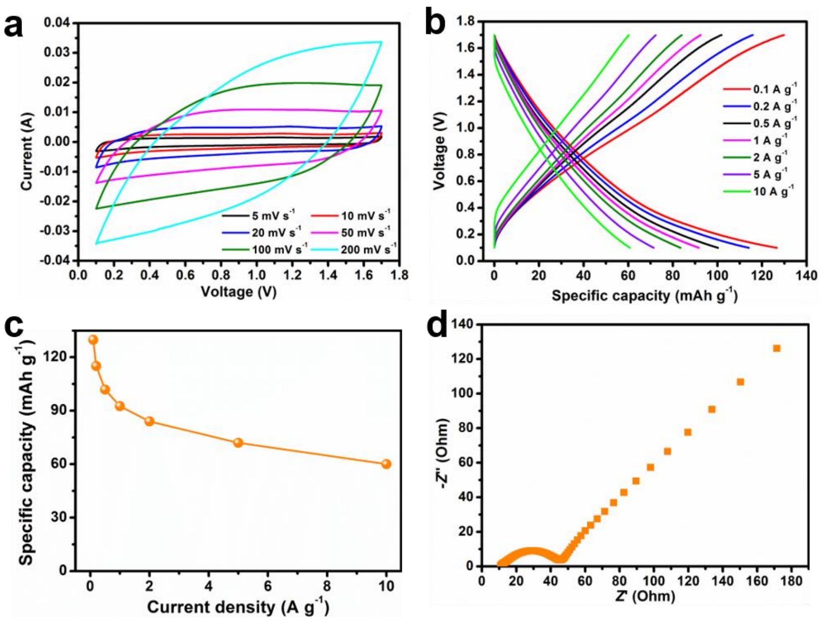

Fig. S6 Electrochemical performances of the ZHC based on commercial activated carbon as cathode (TF-B520//Zn(CF_3SO_3)$_2$//Zn foil): (**a**) CV curves at different scan rates, (**b**) GCD curves at different current densities, (**c**) rate capability and (**d**) Nyquist plot

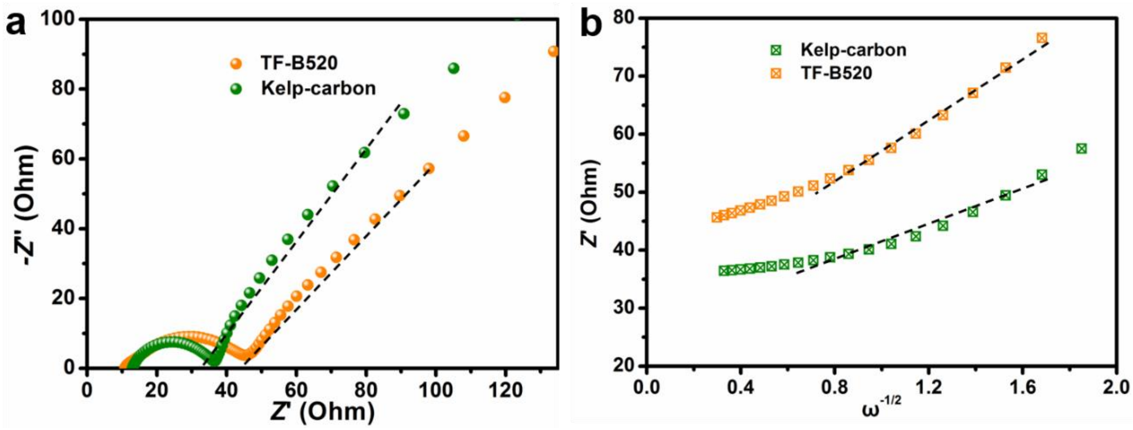

Fig. S7 (**a**) Comparison of the Nyquist plots of aqueous ZHCs based on kelp-carbon cathode and TF-B520 cathode. Dashed lines highlighting the Warburg regions. (**b**) Plots of Z' versus $\omega^{-1/2}$. Dashed lines represent the linear fittings

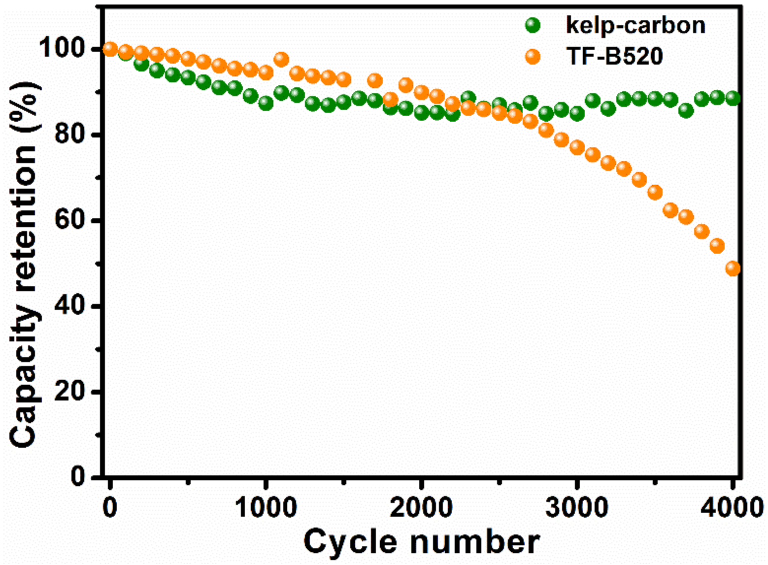

Fig. S8 Comparison on the cycling stabilities of aqueous ZHCs based on kelp-carbon cathode and TF-B520 cathode at a current density of 2 A g^{-1} for 4,000 cycles

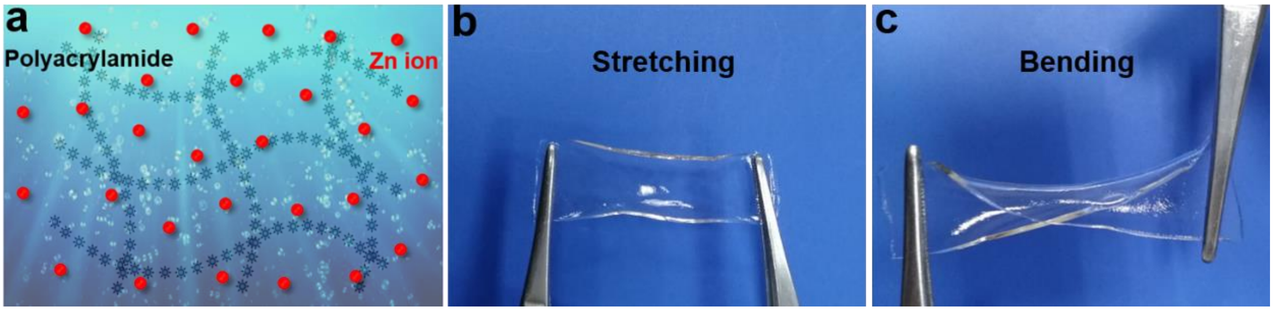

Fig. S9 (**a**) Structural illustration, (**b**) and (**c**) optical images of the $Zn(CF_3SO_3)_2$-PAM hydrogel quasi-solid-state electrolyte

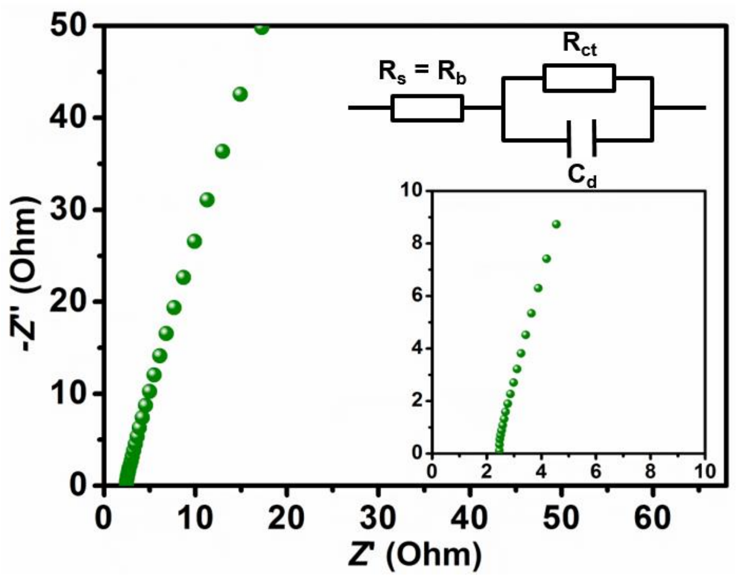

Fig. S10 AC impedance spectrum for the $Zn(CF_3SO_3)_2$-PAM hydrogel electrolyte

The conductivity of the hydrogel electrolyte was evaluated by the electrochemical impedance spectroscopy using a Gamry Interface 1000 electrochemical workstation in the frequency range of 10^{-3} to 10^5 Hz at an AC amplitude of 5 mV. In the measurement, the hydrogel electrolyte was sandwiched between two stainless steel sheets, and the conductivity σ (mS cm^{-1}) was calculated by Eq. (S8),

$$\sigma = L/(R_b \times S) \times 1000 \qquad \text{(S8)}$$

where L (cm) is the distance between the two stainless steel sheets, R_b (Ω) is the bulk resistance (intercept at Z' axis), and S is the contact area (cm^2) between electrolyte and stainless steel sheets. The total conductivity (σ) of the hydrogel electrolyte is calculated to be 12.2 mS cm^{-1}. Electronic conductivity of the hydrogel electrolyte was determined by the direct current (DC) polarization method under an applied DC voltage of 10 mV, which is on the order of 10^{-5} S cm^{-1}, thus the electronic conductivity can be ignored, and the ionic conductivity of the hydrogel electrolyte can be determined to be 12.2 mS cm^{-1}.

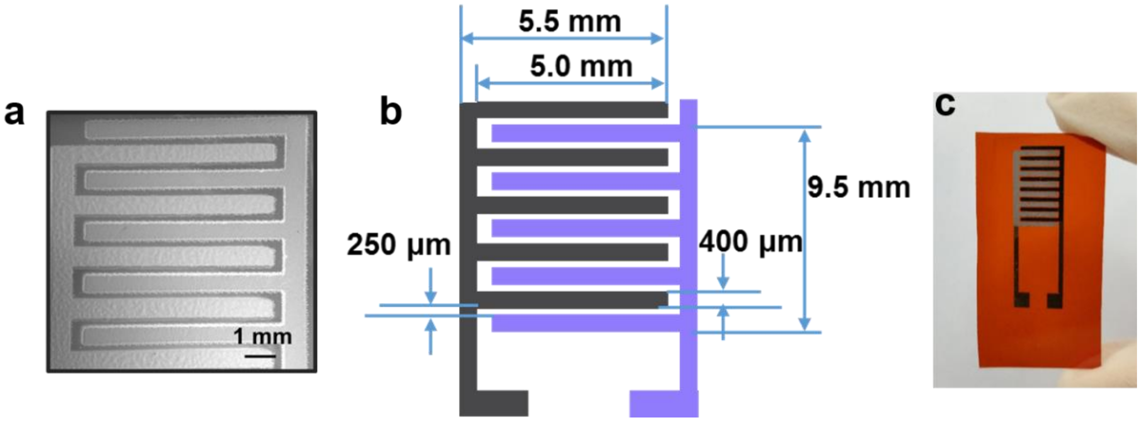

Fig. S11 (**a**) SEM image, (**b**) dimensions and (**c**) optical image of the screen-printed interdigital electrodes

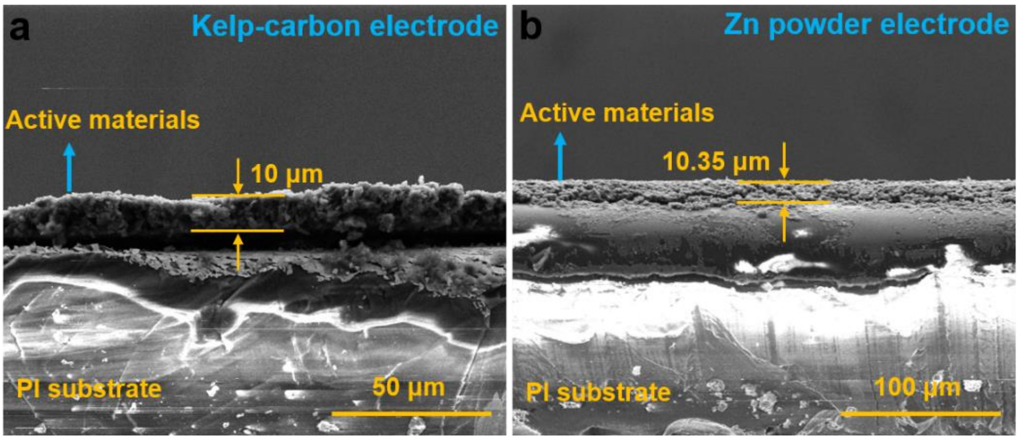

Fig. S12 SEM images on the cross-section of the interdigital electrodes after screen printing (printing one layer): (**a**) kelp-carbon cathode and (**b**) Zn powder anode. The gap between the electrode film and the substrate is caused by the mechanical cutting during SEM sample preparation.

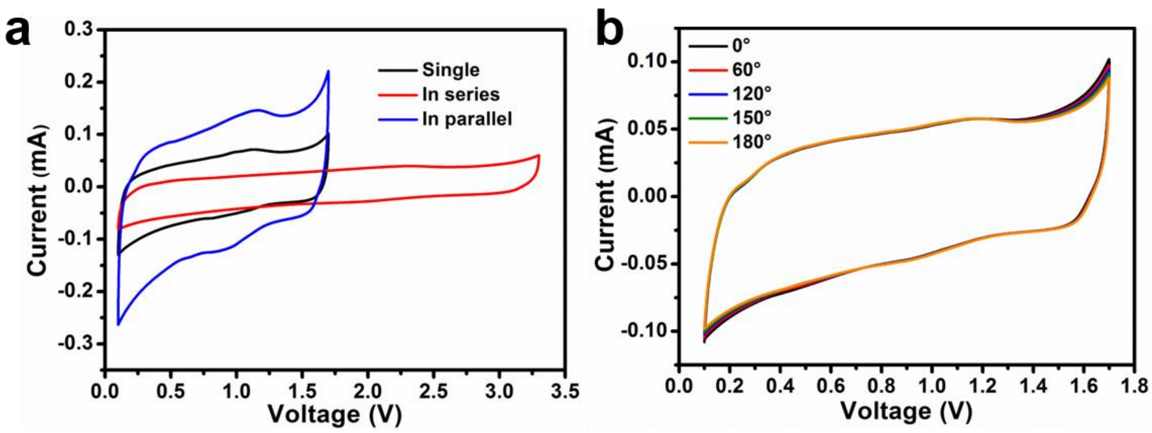

Fig. S13 (**a**) CV curves at 10 mV s^{-1} of a single micro-ZHC, and two cells connected in series or parallel. (**b**) CV curves at 10 mV s^{-1} of a single micro-ZHC at different bending angles

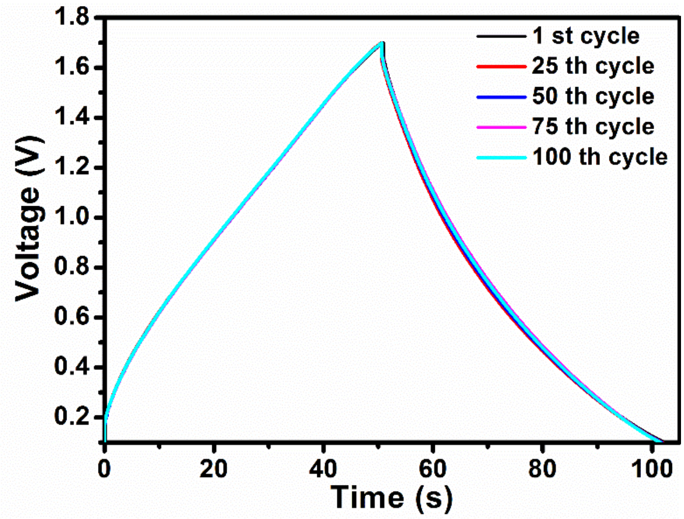

Fig. S14 Typical GCD curves of the micro-ZHC at a bending angle of 120° for 100 cycles

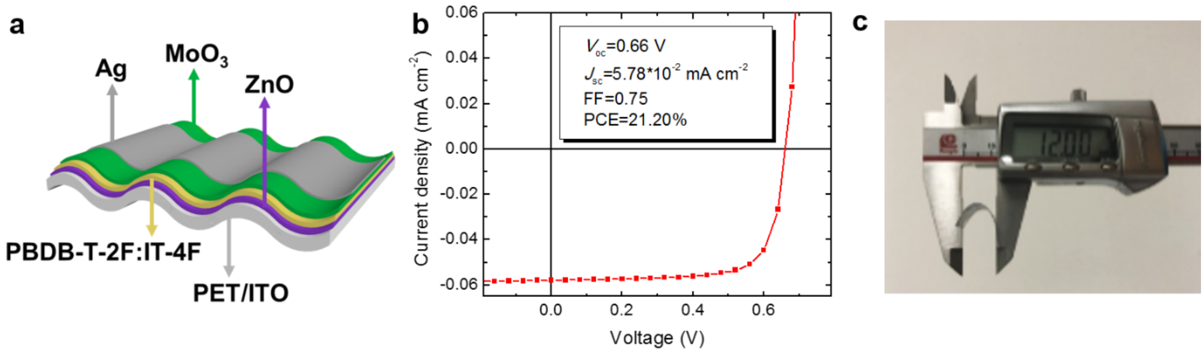

Fig. S15 (**a**) Device structure of the flexible organic solar cell. (**b**) *J-V* characteristic of the flexible organic solar cell at a light intensity of 0.135 mW cm^{-2} (V_{oc}: open-circuit voltage, J_{sc}: short-circuit current density, FF: fill factor, PCE: power conversion efficiency). (**c**) Flexibility of the organic solar cell.

PCE is calculated as follows (power input, Pin = 0.135 mW cm^{-2}):

$$\mathrm{PCE} = \frac{V_{oc} * J_{sc} * \mathrm{FF}}{Pin}$$

$$= \frac{0.66\ V*5.78*0.01\ mA\ cm^{-2}*0.75}{0.135\ mW\ cm^{-2}}$$

$$=21.2\%$$

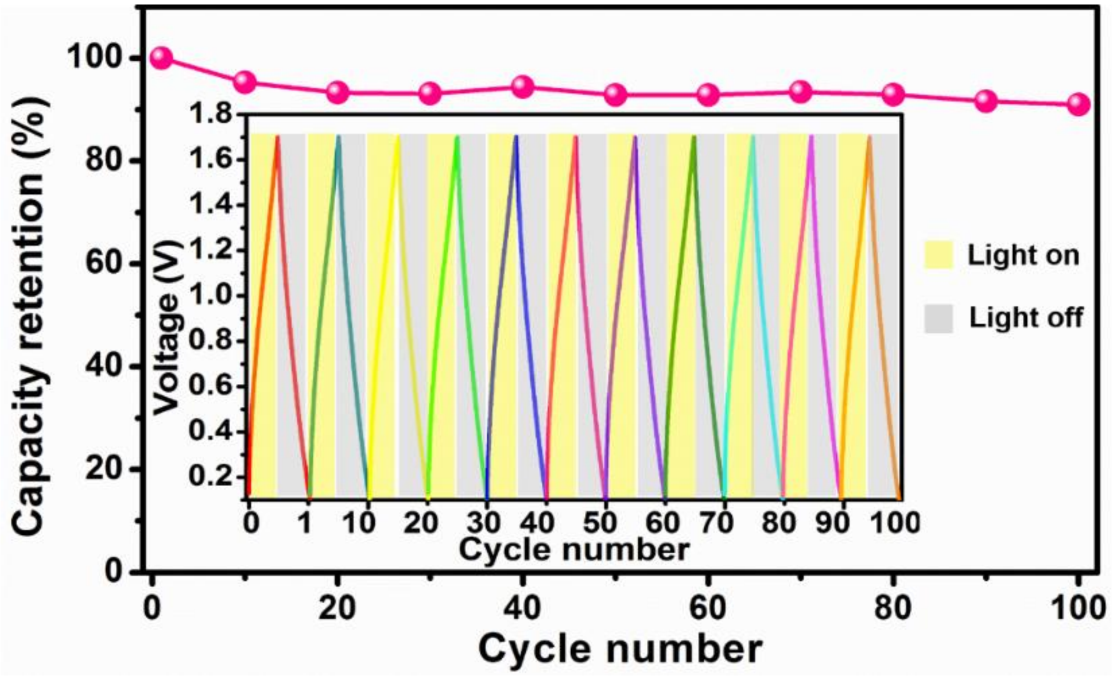

Fig. S16 Cycling stability of the integrated unit at a solar-charging intensity of 4.14 mW cm^{-2} and discharge current density of 2 mA cm^{-2}

Table S1 Specific capacities/capacitances and energy/power densities of the ZHC and Micro-ZHC based on different metrics (including gravimetric, areal and volumetric performances)

ZHC[a]	C_m (mAh g^{-1})	C_m' (F g^{-1})	C_v (F cm^{-3})	E (Wh kg^{-1})	P (W kg^{-1})	E_v (Wh L^{-1})	P_v (W L^{-1})
	196.7	445	284.8	111.5	1300	71.36	832
Micro-ZHC[b]	C_A (μAh cm^{-2})	C_A' (mF cm^{-2})	C_v (F cm^{-3})	E_A (μWh cm^{-2})	P_A (μW cm^{-2})	E_v (mWh cm^{-3})	P_v (mW cm^{-3})
	10.28	23.1	19.7	8.2	40	6.98	34

[a]The gravimetric performance of ZHC is calculated based on the mass of the cathode active material; the volumetric performance of ZHC is calculated based on the cathode volume.

[b]The areal performance of micro-ZHC is calculated based on the total area of the cathode and anode; the volumetric performance of micro-ZHC is calculated based on the total volume of the cathode and anode.

Table S2 Photovoltaic parameters of the single flexible OSC under different light sources (*P*in: power input; *V*oc: open-circuit voltage; *J*sc: short-circuit current density, FF: fill factor; *P*out: power output; PCE: power conversion efficiency)

**Light source**	***P*in (mW cm^{-2})**	**V_{OC} (V)**	**J_{SC} (mA cm^{-2})**	**FF**	***P*out (mW cm^{-2})**	**PCE (%)**
AM 1.5G	100	0.83	18.51	0.69	10.6	10.6
LED	0.135	0.66	0.058	0.75	0.029	21.2
